# Young Peoples’ Construction of DIY Dirt Jumps in Melbourne, Australia, Throughout the Covid-19 Lockdowns

**DOI:** 10.1007/s43151-022-00075-7

**Published:** 2022-05-10

**Authors:** Patrick O’Keeffe

**Affiliations:** Social and Global Studies Centre, RMIT University, GPO Box 2476, Melbourne, VIC 3001 Australia

**Keywords:** Leisure sports, Public space, Mountain biking, DIY urbanism, Youth mental health, Covid-19 lockdowns

## Abstract

Access to public space is critically important for young people, providing key opportunities for self-expression, independence, identity development and relationship building. The Covid-19 pandemic has profoundly affected how young people can engage with public spaces. In Melbourne, Australia, young people have experienced 262 days in lockdown, contributing to escalating anxiety and depression, social isolation, physical health impacts and increased exposure to family conflict and family violence. Throughout this time, there has been a proliferation of young people constructing DIY dirt jumps across Melbourne. This article analyses this unstructured production of public space, focusing on one case study and drawing from council responses. I suggest that through this practice, young people challenge adult interpretations of public space, intentionally or unintentionally, resisting adult control of public space at a time when young people have been denied opportunities for independence and autonomy.

## Introduction

The Covid-19 pandemic has substantially affected the lives of young people across the world (Beames et al. 2021; Magson et al. 2021; Pouso et al. 2020; de Miranda et al. 2020). Within Australia, case numbers of Covid-19 have remained relatively low throughout 2020 and until late 2021 (Li et al. 2021). The policy response to the Covid-19 pandemic has been underpinned by strict social distancing measures, such as school, retail, hospitality and office closures, closure of borders between states and territories and suspension of organised activities such as sport (Li et al. 2021; Loughman et al. 2021; Raynor and Panza 2021; Munasinghe et al. 2020). In cities such as Melbourne, public facilities such as playgrounds, skate parks and sports grounds have been closed for considerable periods of time (Lu 2021; Barnsley 2020).

While strict social distancing regulations and associated penalties are necessary and have undoubtedly saved thousands of lives (Batchelor et al. 2021), they have distanced young people from public spaces at critical stages in their development (Magson et al. 2021; de Miranda et al. 2020). This has had a profound impact on young peoples’ well-being throughout Australia, particularly in Sydney and Melbourne, which have been subjected to lengthy lockdowns (Jagadheesan et al. 2021). People living in metropolitan Melbourne have experienced 262 days under lockdown measures preventing home gatherings, only allowing people to leave home for grocery shopping, caregiving, work (if permitted to do so) and exercise (Zhuang 2021). These measures have underscored the importance of access to public, green spaces, and the unequal distribution of these spaces throughout Melbourne (Oswald et al. 2021).

Young people have been directly and indirectly affected by the Covid-19 pandemic (Beames et al. 2021; Australian Institute of Health and Welfare 2021). School closures have reduced young peoples’ opportunities for socialisation and social development, in addition to difficulties and inequalities associated with online education (Magson et al. 2021; de Miranda et al. 2020; Jones et al. 2020). Confinement to homes has increased young people’s exposure to family violence, while also exacerbating conflict with family members (Loughman et al. 2021; Power et al. 2020; Jones et al. 2020). Financial impacts of the pandemic have caused widespread job losses and underemployment, with young people highly represented among casual employees in industries where job losses have been greatest (Biddle et al. 2020). While there is clear evidence of young peoples’ resilience in the face of multiple challenges (Beames et al. 2021; Magson et al. 2021), young people have exhibited increased instances of anxiety, depression and isolation (Batchelor et al. 2021; Jagadheesan et al. 2021; Li et al. 2021; Loughman et al. 2021; Oswald et al. 2021; Power et al. 2020). Associated with mental health impacts, young peoples’ physical health has also been adversely affected as opportunities for exercise and contact with nature have been suppressed (Li et al. 2021; Oswald et al. 2021).

This research observes young peoples’ claiming and shaping of a public space, focusing on the construction of dirt jumps in a suburban park in the outer Eastern suburbs of Melbourne. This research draws together themes and concepts studied in cultural geography and youth studies research examining young people and public space. Production of this space was primarily studied through the physical changes young people made within the space. Photographs and field notes were taken to capture these changes. This observation and analysis draws on Vivoni and Folsom-Fraster (2021), Vivoni (2013) and Frers and Meier (2017), who have conceptualised changes to place as observable through traces that people leave through their spatial production. In relation to the impacts of the Covid-19 pandemic upon young people, I examine dirt jumps, as traces which enable communication, collaboration and solidarity between young people, and demonstrate resistance to adult control of public space. This research aims to understand the significance of DIY dirt jumps created by young people as a form of spatial production during periods of lockdown in suburban Melbourne, during the Covid-19 pandemic. In addition, through analysing policy documents produced by council in response to the proliferation of dirt jumps, this study addresses how councils have responded to this expression of power and resistance by young people.

## Methodology

For this research, I observed the construction and use of dirt jumps by young people in a public park in an outer-Eastern suburb of Melbourne. The primary observations obtained throughout this research were of changes to landscape, through the continual development and redevelopment of dirt jumps and short mountain biking paths. As the lockdown restrictions limited time spent exercising in public spaces, the park was often sparsely populated when I was there. The dirt jumps communicated activity, though I only observed people either using or creating these structures on a small number of occasions.

Through observing this pattern of use, I draw on the concept of traces as indicators of young peoples’ engagement with place (Vivoni and Folsom-Fraster 2021; Hollett and Vivoni 2020; Frers and Meier 2017). In relation to street skateboarding, Vivoni and Folsom-Fraster (2021, p. 313) describe traces as a means of communicating the alternate production of a public space:

As skateboarders drift through the city, traces remain as evidence of an alternative use of the built environment. Skateboard scuff arises from an unspoken collective effort. Filth traces adorn concrete surfaces as both markers of previous use and harbingers of unscripted encounters in public space. Candle wax and board art coalesce on granite ledges as skateboarders glide across varied surfaces. Skateboard scuff markings are works-in-progress, smudge palimpsests and friction regulating technologies. Like graffiti writers, street skateboarders make lines through a series of manoeuvres and leave traces that signal unprescribed uses, meanings, and pleasures.

As Vivoni and Folsom-Fraster suggest, traces left by young people in public spaces, often through participation in ‘illegal’ practices, provide a means of observing young peoples’ reproduction of public space, and altering of this space to reflect young peoples’ agency and resistance to adult control. This is indicated through Vivoni and Folsom-Fraster’s (2021, p. 313) conceptualisation of wax and scuff marks left behind by skateboarders in ‘found’ skate spots:

If left unused, scuff marks tend to dry-up. Pieces of candle wax are often left behind as gestures of solidarity. The clandestine upkeep of a scuffed skate spot is a communal practice that entails discretion and fine-tuning.

This reflects a communication between different groups of skateboarders, through their production of public spaces, as sites of play and also resistance and solidarity among (often, though not exclusively) young people as subversive users of these spaces. As Frers and Meier (2017) describe, traces provide a key expression of resistance which remains when young people have left the space. In this way, Frers and Meier (2017) conceptualise acts of resistance, such as street skateboarding in a public space, as having resonance beyond the moment in which this act is taking place. According to Frers and Meier (2017), the traces left behind as a result of this act, such as scuff marks or candlewax produced through skateboarding or dirt jumps created by mountain bikers, reflect the afterlife of that act of resistance.

As I observe in this paper, young peoples’ changes to the landscape in Pine Tree Park can be understood in terms of communication, as well as solidarity among other young people and bike riders, and as a lasting expression of resistance against adult control over private space. The dirt jumps as traces which represent communication, solidarity and resistance are particularly significant at a time where young peoples’ mental and physical health was substantially affected by lack of access and control over public spaces.

### Observing and Photography ‘Dirt Jumps’ as Spatial Production

The park where I conducted my observations is 1.5 km from my home in Melbourne’s outer Eastern suburbs. The case study is a hilly, medium-sized park in an outer-Eastern suburb of Melbourne, approximately 25 km from the Melbourne CBD. Throughout this study, I refer to this park as ‘Pine Tree Park’. These unstructured observations were conducted during daily, mid-morning visits to the case study location during Melbourne’s 6th lockdown (from August 6 to October 22, 2021), through my position as an adult and outsider, who did not participate in dirt jump construction or use.

When at the park, I observed changes to the landscape, primarily through the traces left by young people using this space. My observations focused on identifying the construction of new jumps or tracks, the increasing size and gradient of the jumps, modifications to existing tracks, the materials used to construct the jumps and tracks and, in some instances, the tire marks to indicate the type of vehicle ridden over the tracks. I also observed the aftermath of suppression of this activity, noting at times how jumps had been demolished, presumably by heavy machinery, as indicated by tracks left in the ground. I recorded these observations as notes and photographs, which provided a visual representation of the changes to the space. I did not take photographs when young people were present in the immediate area (although one photograph features a bike, I could not see the owner of the bike at the time).

When young people were present, I observed how they shaped the space, including planning and construction. I observed groups of two to three young people constructing jumps with shovels and other implements. Regarding play within this space, I observed how the jumps and tracks were used. However, the observations of young people present in the space were rare and often fleeting. As I was also subject to restrictions on my activities, I did not ever stay in the space itself for more than a couple of minutes. This meant that my observations of young people reflected snapshots of engagement and use. This highlights the value of photographs of the space in this instance, which provided a lasting, detailed recorded which can be observed at greater length. The research was conducted spontaneously, to analyse an important process at a very specific moment in time, as a research and teaching academic with an interest in young peoples’ creative and subversive production of public space.

Coupled with these observations, I include analysis of documents produced by councils in metropolitan and regional Victoria, which have addressed the issue of spontaneous dirt jumps in respective Local Government Areas. The purpose of this part of the analysis is to consider Council responses to construction of DIY dirt jumps, through their role as managers of these spaces. In particular, these documents are analysed to consider questions including the following:

- How are spontaneous, DIY builds conceptualised by Victorian local councils in policy documents responding to the construction of dirt jumps by young people?
- How have Victorian local councils responded to young peoples’ construction of DIY dirt jumps?
- How are young people included in decision-making processes concerning the production of public spaces aimed at facilitating youth leisure activities?

Drawing on King and Church’s (2020) analysis of young people participating in independent, ‘wild builds’, this research analyses the extent to which these practices and permitted in council strategies on public spaces.

### Young People and Public Space

This article now draws together literature analysing young peoples’ interactions with public space, conceptualising young people as marginalised participants in public space and as agents of resistance through subversive practices. I then explore literature on youth practices which shape public space, such as skateboarding and mountain biking. This further analyses the meaning and significance of these activities, and then how this might be understood in relation to my observations of dirt jumps constructed by young people during lockdown periods.

Public space provides young people with relative independence and autonomy, away from the home and schools, where parents, caregivers and teachers are recognised as authority figures (Wijntuin and Koster 2019; Wood et al., 2014; Rannikko et al. 2016). Public spaces are critical in young peoples’ development, particularly in relation to identity formation, relationship building and socialisation and exploration of their emerging independence (Pederson and Gram 2018; Cuervo and Wyn 2017; Gilchrist and Wheaton 2017; Baker 2015; Pickering et al. 2012). However, suspicion of young people, and limited opportunities for autonomous play and activity outside of ‘child’ spaces such as playgrounds, reduces opportunities for engagement with public space and marginalises young people as participants in public space (Carroll et al. 2019; Woolley et al. 2011).

Valentine (1996) has suggested that public space can be more accurately conceptualised as ‘adult space’. Across different Western societies, adults have sought to the defined rules, behavioural norms and expectations, and values which govern how public space should be used (Carroll et al. 2019; Nolan 2003; Matthews et al. 2000). In these ‘adult spaces’, young people are conceptualised as a ‘deviant other’, who are disrespectful, recalcitrant, associated with criminal acts and disruptive to dominant, adult uses and experiences of public space (Woolley et al. 2011; Nolan 2003; White 2001). For example, young people engaging in leisure activities such as BMX riding, skateboarding and mountain biking are frequently portrayed as anti-social, delinquents and vandals who contribute to social disorder (King and Church; Brown 2016; Taylor and Khan, 2011; King 2010).

Young people’s occupation of public space is considered as a threat to the adult hegemonic control of public space, and the private property rights underpinning ownership of public spaces in capitalist societies (Vivoni and Folsom-Fraster 2021; Chiu and Giamarino 2019; Howell 2008; White 2001). Rather than having an equal right to use public space, young people are portrayed by adult spatial managers as requiring surveillance, policing and deterrence (Carroll et al. 2019; Baker 2015; Wood 2015; Woolley et al. 2011). This involves measures criminalising young peoples’ practices in some public spaces, including ‘hanging out’, skateboarding and bike riding (Brown 2016; Taylor and Khan, 2011; Woolley et al. 2011).

While adults, as the owners and managers of public space, can deploy significant resources in attempts to police young people, it would be incorrect to assume that young people are powerless in this relationship (De Backer et al. 2019; Wood 2015). In claiming, appropriating and occupying public space, young people express their power and resistance to adult control (De Backer et al. 2019; Rannikko et al. 2016; Wood 2015). Throughout the Covid-19 pandemic, and periods of tight social distancing restrictions in particular, young peoples’ capacity to express power in public space becomes increasingly significant. While authorities and institutions have broadly expressed concern for young people’s well-being, policy concerning young people is frequently made in their absence (Bessant et al. 2021). Furthermore, negative constructions of young people as disrespectful and lacking civic responsibility were mobilised on numerous occasions to blame young people for public health transgressions, contributing to skate park closures (Barnsley 2020; Fraser Coast Regional Council 2020).

### ‘Dirt Jumps’ as a Spatial Practice

The spontaneous, independent construction of dirt jumps by young people has been examined in relation to temporary mountain bike trails in peri-urban and urban environments throughout the UK (King and Church 2020, 2015), and DIY BMX parks featuring pump tracks and dirt jumps in the USA (Olsen 2021; Smith 2021). King and Church (2020, 2015) and King (2010) have examined young mountain bikers’ practices of creating ‘wild builds’ or ‘secret spots’, incorporating trails and dirt jumps in locations which are relatively hidden from adults’ surveillance and intervention. King and Church (2020) and King (2010) suggest that the practice of mountain biking is integral to participants’ sense of identity, while helping young people overcome isolation. The practice of building jumps and trails provided young people with a sense of independence, autonomy and decision-making power (King and Church 2015; King 2010). This contrasts with young peoples’ experiences of marginalisation in ‘dig days’ organised by mountain biking clubs, where young people were less able to participate in key decisions (King and Church, 2020; King and Church 2015, p. 291). Conversely, while unsanctioned ‘wild builds’ organised independently by young people frequently lead to conflict with landowners, this practice enabled young people to participate in an unstructured activity where they had full control and voice (King and Church 2015).

According to King and Church (2020, 2015), this preference for unstructured activities and spaces is directly related to the ways that they were able to engage with the places they were developing. This emphasises the importance of spaces that inspire young people’s independence, which they are able to create without adult intervention (Sand & Hakim-Fernandez 2018; Sand 2017). This freedom is not always afforded to young people through more structured programs designed by adults, which can unintentionally marginalise the ‘practices and places that young people develop for themselves’ (Sand 2017, pp. 288–289). As such, institutional places like schools, sports clubs and youth-focused organisations are often portrayed as the legitimate sites of youth culture (Sand 2017). Similarly, state- and corporate-funded ‘youth’ spaces can also contribute to a form of passive marginalisation, where practices and activities are able to be packaged and controlled by adults, and delivered to young people (King and Church 2020).

### Claiming, Poaching and Appropriating Spaces in ‘Leisure’ Sports

In a context where young people are frequently marginalised and disempowered in public space, spaces created by young people, however minor, can convey a sense of power and control within urban environments (Carroll et al. 2019; Sand 2017; Frers and Meier 2017). As De Backer et al. (2019, p. 246) describe in relation to negotiating power in public space, ‘visible and invisible minor acts’ can be greatly significant. For example, street skateboarders ‘claim their right to the city’ through their engagement with, and capacity to shape, challenge and reinterpret public space (Glover et al. 2021, p. 44). This involves the appropriation and reinterpretation of public spaces designed for adult uses as autonomous play zones, often through unsanctioned modifications (Glover et al. 2021; Vivoni 2013, 2009). Fundamentally, practices such as street skateboarding shift the perception of cities as sites of production to a site of play, allowing for the playful reimagination of mundane landscapes (Vivoni and Folsom-Fraster 2021; Chiu and Giamarino 2019; Nolan 2003).

As Vivoni (2013) mentions, developing such spaces, and such an active, visible presence of young people in public space, requires collective work which is mostly uncoordinated. Similarly, Smith (2021) highlights the ‘dis/organisation’ among BMX rider-builders contributing to the maintenance of a DIY BMX park. Smith (2021) describes the fluid organisation whereby the young people build the space along with others, though not in the coordinated, structured approach evident in managed builds described by King and Church (2020, 2015).

This leads to the process of constructing DIY spaces, with Hollett and Vivoni (2020) describing ‘making’ as a socio-political process. Hollett and Vivoni (2020) suggest DIY skate parks are a form of subversive creativity, similar to graffiti and guerrilla gardens, which involve the:

Temporary and illegal appropriation and physical and constructional modification of forgotten spaces in the city (Peters 2018, p. 207, cited in Hollett and Vivoni 2020, p. 3).

For skateboarders and bike riders, the ‘making’ of spaces through re-interpreting and augmenting physical environments may be as significant as the actual play within the space (Olsen 2021; Hollett and Vivoni 2020; Regener 2019; Gilchrist and Wheaton 2017). DIY spaces such as ‘wild builds’ and DIY skateparks can foster deep social connections, through the collective (albeit, often fluid and uncoordinated) process of making the space (Regener 2019). This sense of meaning can be further understood in terms of the collective response to a social issue or problem, and the contribution which is made to a population which might benefit from the work.

## Case Study: Pine Tree Park

Pine Tree Park consists of walking trails connecting the different sections of the park, with two different playgrounds, an area of native grassland including a number of orchids and a large number of tall pine and eucalypt trees. Nearby one playground, there is an area consisting of undulating ground underneath large pine trees. This area also features large bluestone rocks, native grasses and young eucalyptus trees. Previously, this area has not been widely used by people in comparison with the walking trails and playgrounds in the park.

The undulating aspect of this area in many ways resembles a natural, albeit small, mountain biking course. During Melbourne’s 6th lockdown, this site became popular among young bike riders, who augmented the existing structure of the space to create a small mountain biking trail featuring dirt jumps.

**Image 1:**
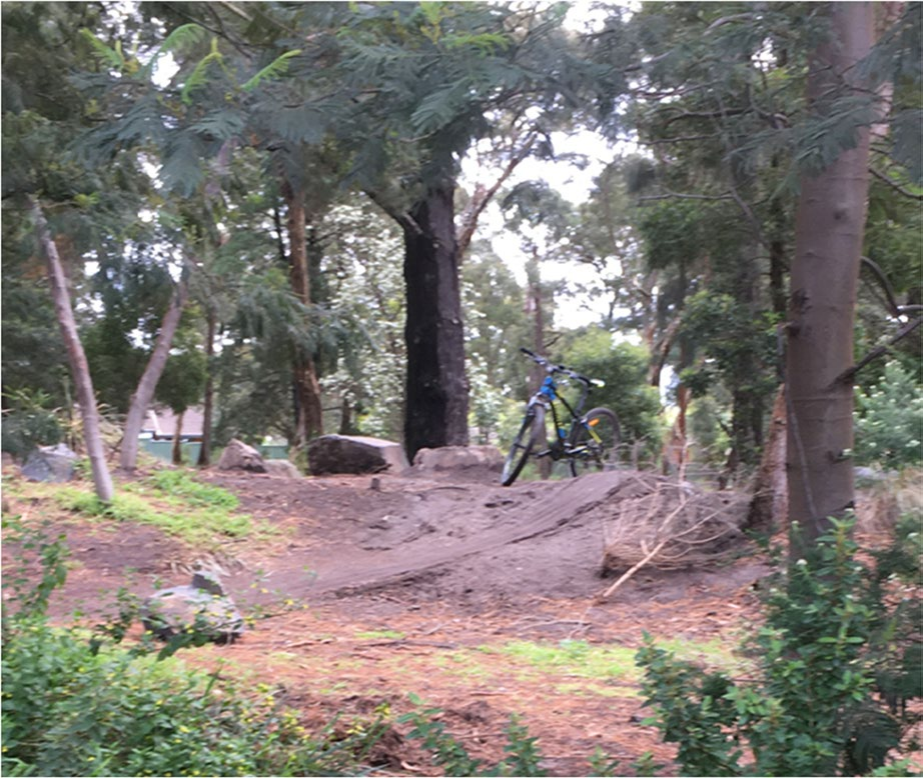
Dirt jump constructed in park. October 1, 2021

Initially, I observed small groups of two to three young people constructing jumps in this spot with a shared shovel. Image 1 shows the first jump created, which is the central part of the course. Within days of the initial jumps being created, groups of up to 15 young people were riding in this space, taking turns to complete the course and filming each other’s jumps with smartphones. These groups were not associated with the initial rider-builders, and appeared to be disparate collections of young people sharing the space. Throughout the next 2 weeks, new jumps were added to the course by new rider-builders. During this time, there was no evidence DIY construction in other parts of the park.

I frequently observed changes to the landscape, without necessarily observing young people making these changes. Each day the tracks changed in some way, becoming more complex and interconnected and featuring steeper jumps and new jumps. While young people were rarely present, I could observe the development of their work as a visible reminder of the consistent, collective effort towards shaping and moulding a fun landscape. In the absence of young people, the dirt jumps and tracks were the traces remaining of young peoples’ play in the space, and of the capacity of young people to exercise power in public spaces. As traces of young peoples’ activity, the dirt jumps and tracks can be understood as a form of communication, a collaboration between groups of young people who were not connected through other means, as an expression of solidarity among young people and of resistance to adult control of the space.

First, the traces provided a means of communicating the repurposed nature of the space. This communication can be received by adults, indicating the presence of young people as agentic participants in the space. However, the dirt jumps also provide a means through which young people communicate with each other, at a time when social interaction is restricted. The dirt jumps not only reconfigure the space as a place of play, but also communicate this message to other young people. The DIY nature of the jumps also communicates to young people that they also have the capability to participate in this production of a place of fun and play. Constant adaptations to the space communicate to fellow users, through changes in shape and materials.

In this way, ongoing construction of the space highlighted a collective effort to adapt and improve the dirt jumps and tracks. While young people were restricted through capacity to socialise, the collaborative though uncoordinated effort to produce the space revealed a shared willingness to participate in a positive activity with other young people. The dirt jumps, as traces, revealed and communicated the ongoing desire of young people to be involved in this shared project. This leads to the solidarity among young people, revealed through the changes to the landscape, in the traces that each small group left behind. Throughout the Covid-19 pandemic, young people have experienced social isolation, adverse impacts on mental health and limited opportunities for physical activity. The shared construction of the jumps can be understood as an expression of solidarity among young people, and a desire to support others through their labour, by creating a place of fun, play and exercise.

**Image 2:**
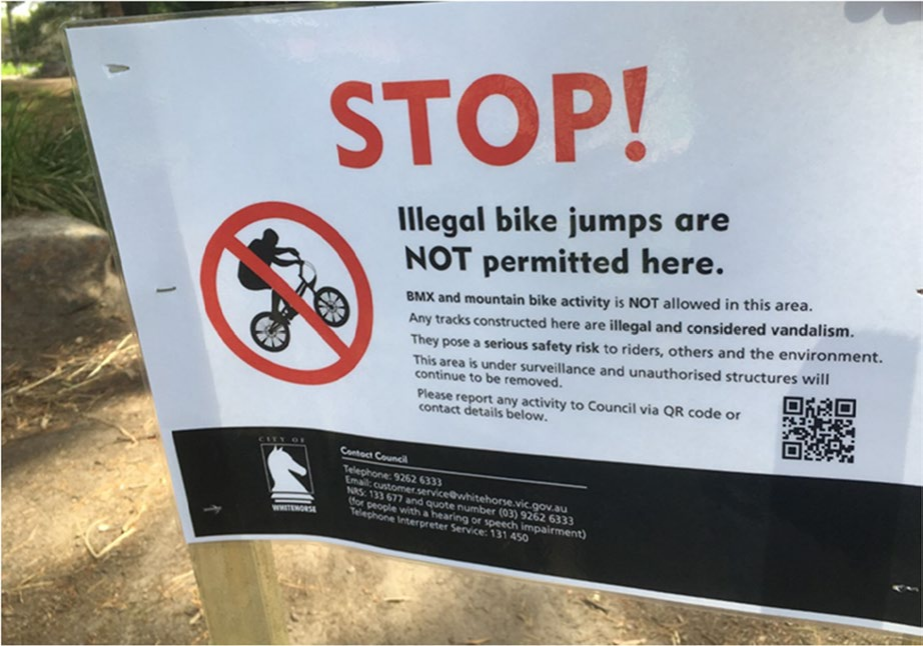
Sign at this location compelling rider builders to stop creating dirt jumps. October 9, 2021

Following this period of intense construction and use, council employees began to routinely demolish the jumps. On two occasions, I witnessed compact bulldozers flattening jumps. This response did not seem to be in proportion to the extent of the modifications to the space, which were relatively modest. In one instance, the initial rider-builders were present and constructing jumps when council employees arrived with the bulldozer. The employees told the young people to cease their work. The young people left, and the bulldozer was then used to dismantle the jumps. The sign in Image 2 was then erected, informing rider-builders that their work was ‘illegal and considered vandalism’ by the council. As Image 2 shows, the council note warns that BMX and mountain bike riding was ‘NOT permitted’ in this space, which was under ‘constant surveillance’. Whereas signals communicated by young people through traces left in the landscape was subtle and unspoken, communication by adults managing these spaces was direct in its reiteration of adult-imposed rules governing public space, and violent demolition of dirt jumps with heavy machinery.

**Image 3:**
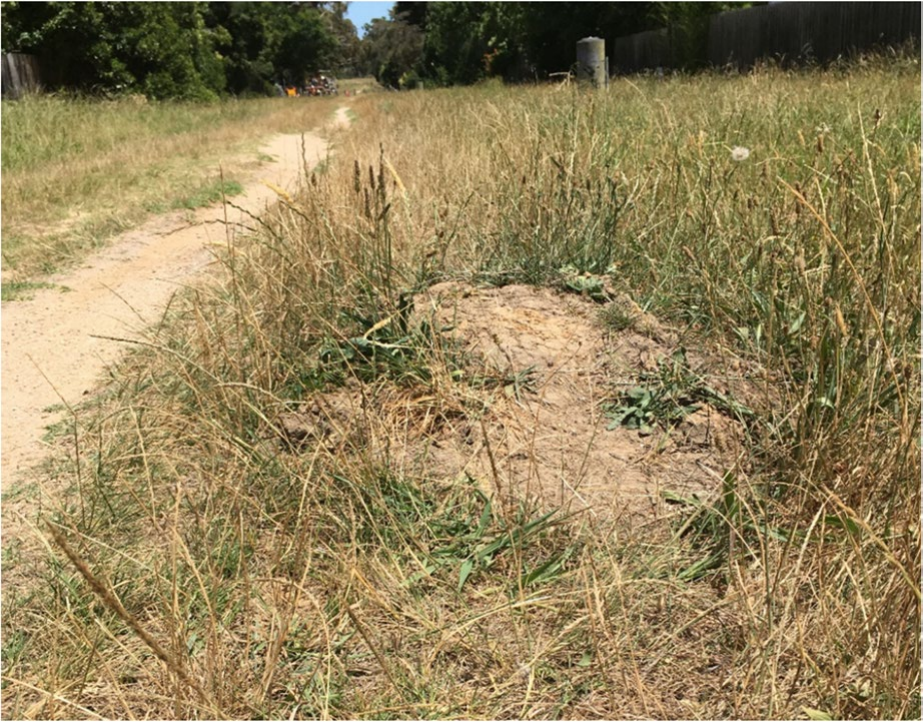
Dirt jump constructed during lockdown in mid-2021, overgrown with grasses and weeds by early 2022

Initially, young people resisted the demolitions and signage by rebuilding dirt jumps. However, the intensity of the council response aimed to counter young peoples’ resistance, with jumps that had taken effort and time to create being bulldozed in a very short space of time. While one or two jumps remained and continued to be used by noticeably fewer young people, concerted digging efforts in this location ceased. Jumps were created in new locations nearby, including alongside pathways (Image 3). As young people returned to school with the end of the 6th lockdown in Melbourne, this also led to a shortage of labour, and perhaps a reduced need to communicate through traces in the landscape and a reduced need to access alternate spots of fun and play.

The remaining jumps were not frequented by large groups of people, and the lack of maintenance on these jumps suggests that there was no ongoing occupation of these new sites. However, the remains of these jumps continue to provide trace reminders of young people’s transformation of these spaces. This also provides a lasting indication of young people’s resistance to adult control of public space during the Covid-19 pandemic in Melbourne, and shared solidarity in creating a project that might provide a level of support and comfort throughout this very difficult period of time.

## Dirt Jumps Throughout Melbourne

Examining council responses to DIY dirt jumps is important in understanding how young people’s spontaneous production of public space is framed by the managers of that space. This response is significant in relation to young people’s capacity to subvert adult control of public space, and also in relation to the willingness of authorities to defer a level of ownership and control to young people as decision makers in public space. Similar to Pine Tree Park, councils in other areas across Melbourne responded by demolishing jumps due to safety and environmental concerns (City of Moonee Valley 2021a; Manningham Council 2021; City of Whittlesea 2021). While acknowledging the creativity and enthusiasm shown by young people, councils have framed these actions as ‘illegal’ or ‘unauthorised’ (Nillumbik Council 2020). In turn, many councils across Melbourne, including outer metropolitan areas, have initiated consultation processes with community members in relation to dirt jump constructions (City of Moonee Valley 2021a; Manningham Council 2021; Mornington Peninsula Shire Council 2020; Nillumbik Council 2020). In these examples, residents were encouraged to participate in the consultations through various means, and register their interest for contributing towards planning of these facilities (Nillumbik Council 2020). Consultations have intended to lead towards council strategies for developing spaces that meet young people’s needs, such as professionally constructed BMX and mountain bike tracks. This article now considers strategies developed by two councils in metropolitan Melbourne.

### Nillumbik Council: BMX in Nillumbik

The Nillumbik Council (2020) consultation report is based on a survey completed largely by young people. This survey centres on the use of existing facilities, potential limitations of existing facilities and possible upgrades and the development of a new BMX park at a location identified by council. For example, the survey asks (Nillumbik Council 2020, p. 20):

Council are exploring the opportunity to expand one of our existing park areas to incorporate BMX and other outdoor recreational activities for youth. Select your preferred option:

- Option A) upgrade Eltham Skate Park
- Option B) upgrade Alan Marshall Reserve, Research
- None of the above

This survey compels respondents to vote for potential developments of new and existing facilities, with the alternative response being no development of any facilities. There is no option to suggest retaining wild builds completed by young people. Given that the consultation is intended to address the proliferation of DIY dirt jumps and tracks, the omission of questions which seek to understand and support this practice is notable. The survey does not address spontaneous construction of dirt jumps in any way, while the consultation report repeatedly states that they are unauthorised, unsafe and harm environments. The report’s key recommendation is to develop a funding application to the Victorian Government’s Park Revitalisation Grants scheme to upgrade this facility (Nillumbik Council 2020, p. 4).

The report implies that any decision making around any facilities will be made by local and state governments, rather than young people in the area, despite noting that (Nillumbik Council 2020, p. 4):

It is important for any future work to include the ongoing engagement and consultation with the users of these facilities and the broader community to ensure young people continue to be engaged in matters that affect them and have a voice in the issues they are passionate about.

However, this still centres decision making and authority with the council. Through neglecting to focus on DIY dirt jumps in the survey underpinning this consultation, the council has suggested that this is not an issue which young people can engage the council with.

### City of Moonee Valley: Young People Outdoors

Similarly, the City of Moonee Valley consulted young people on their use of public spaces, through consultations at facilities and an online survey. In contrast to the Nillumbik Council, City of Moonee Valley (2021b, p. 5) surveyed young people on DIY tracks, asking:

- Do you use unplanned tracks in Moonee Valley?
- What do you get out of using the dirt tracks/jumps? e.g. socialising, exercise

Answers to the first question suggested that a large percentage of respondents used DIY tracks (City of Moonee Valley 2021b, p. 13). Responses to the second question significantly preferenced socialising and exercise, though it is difficult to determine the extent to which the question prompts influenced this outcome (City of Moonee Valley (2021b, p. 13). The City of Moonee Valley (2021b) claims that these responses suggest that the primary value of the DIY builds is therefore socialising and exercise, arguing that the youth-led nature of these builds is insignificant. This is used to argue against permitting DIY builds, claiming that council-built facilities can meet the claimed primary purpose of exercise and socialisation.

## Discussion

Young people experience ongoing exclusion from public space, accentuated by Covid-19 social distancing restrictions, and are not genuinely included in discussions which have informed policy responses to Covid-19 (Jones et al. 2020). In addition, young people have experienced significant increases in mental ill health, disrupted education and increased exposure to family violence and are among the population groups who are most anxious about the implications of the virus itself.

In this context, the proliferation of dirt jumps across Melbourne can be understood in multiple ways. ‘Dirt jumps’ can be interpreted as an expression of power and control over place, in an environment where young people have been systematically disempowered. Where policymaking and discussion have been about young people, though not ‘with’ young people (Jones et al. 2020), ‘dirt jumps’ could be a means through which young people emplace themselves within urban environments. This practice can also be considered as an ‘active coping strategy’ as described by Beames et al. (2021, p. 6), combining socialising, hobbies, physical exercise and contact with nature, which are most strongly associated with better mental health outcomes through the pandemic (Oswald et al. 2021), as a practical response to anxiety, depression and social isolation, through creating places which foster social interaction, play and exercise. In addition, the widespread nature of this practice throughout Covid-19 lockdowns suggests that young people recognise the value in creating and shaping places, free from the intervention of adults.

Confinement to homes impacts all members of the population, though for young people, who have less authority and power within the home than their parents, and who depend on access to public spaces, this raises considerable challenges. Creating ‘dirt jumps’ with others, through reimagining and reshaping urban landscapes, enables young people to be creative and make places which inspire spontaneous play, and allows young people to develop a sense of agency in local environments, at a time of intense uncertainty, anxiety and stress. These practices, I suggest, entail a form of active participation in society (Oswald et al. 2021), which addresses a social issue affecting young people, at a time when young people were largely erased from discussions around their well-being. As traces left behind in the landscape, DIY dirt jumps provide an important means of communication between young people. The dirt jumps highlight the reproduction of these spaces as places of play, while also communicating to young people that they can shape their local environment. The jumps communicate the collaborative work done by young people in reimagining the space. Communication occurs between different young people who are otherwise not connected, through their reshaping and development of the space. The traces also communicate young people’s resistance, solidarity and willingness to develop a sense of autonomy within public space.

While the City of Moonee Valley (2021b) downplays the significance of young people constructing their own jumps; in light of the Covid-19 lockdowns and reduced agency within public space this practice should be considered meaningful. Studies exploring young people’s DIY skateboarding and mountain biking spaces have suggested that the process of making has significance in itself (King and Church 2020; Regener 2019), and literature on young people’s production of public space further illustrates the importance of being able to shape spaces young people inhabit. I suggest that young people building dirt jumps are reframing themselves as active citizens, rather than passive consumers of services provided by adults. To a significant degree, council consultation processes implemented in 2020 and 2021 seek to restore power relations that undermine young peoples’ ability to actively engage in public space.

Council concerns are well-founded in relation to instances of ecological damage caused by dirt jumps created in sensitive and revegetated areas, as young people may not recognise the environmental significance of these areas. However, rather than seeking to support young people’s agency in public space in ways that also support natural environments, councils have focused on criminalisation of dirt jumps and mountain biking in these places. This construction is underpinned by a framing of uncontrolled young people as inherently deviant and disruptive, whose shaping of public space amounts to vandalism.

The council consultation strategies included in the article outline mechanisms for including the voices of young people in constructing dirt jumps and tracks. However, this strategy reflects a form of passive marginalisation, where councils are co-opting spontaneous practices developed by young people (King and Church 2020; Sand 2017). Young people’s DIY builds are banned through these strategies, with any future work completed through formal processes managed by councils. First, this removes the opportunity for spontaneous and transformative play that has been necessary during short- and long-term lockdowns where council facilities have been closed (Carroll et al. 2019). Second, this misunderstands the value for young people actively participating in the construction of dirt jumps, and reshaping public space (Olsen 2021; King and Church 2020, 2015). Through this work, young people express their power, independence, autonomy and ownership over public spaces, engage in meaningful work and benefit from the social aspect of creating the space (Olsen 2021; King and Church 2020, 2015; Regener 2019). Council responses reduce young peoples’ capacity to participate actively in society as decision makers, with decisions, resourcing and work largely performed by officials and professionals.

## Conclusion

Through spontaneously building dirt jumps, young people responded to the various issues they confronted during Melbourne’s lockdowns, as a practical response to a social issue. Where young people had been routinely distanced from discussions around their well-being, they developed a solution to concerns such as lack of exercise, isolation and mental ill health with their own resources, without help from adults. In doing so, young people created landscapes which enabled them to experience unstructured play. Rather than recognise the depth of young people’s responses to an escalating mental health issue, policymakers and councils continue to perceive young people as a population which threaten their management and control of public space.

The practice of creating DIY dirt jumps can also be understood as a means through which young people imprint themselves on the landscape. This signalled to adults and other young people an intent to creatively reimagine public space, and also resist the adult domination of public space during lockdowns in Melbourne. Analysis of council responses to DIY dirt jumps reflects an intent to engage young people in the conceptualisation and design of public space, though in ways which reinstate adult hegemony within public spaces. In policy documents included in this study, young people are framed as consumers of public space, rather than as autonomous actors with genuine power to reshape this space. This article builds on research exploring the conflict between adults and young people in public space, while contributing to understanding of DIY dirt jumps as a phenomenon which emerged across Australian cities and regional towns throughout the Covid-19 pandemic.

Further studies seeking to understand the significance of DIY spaces created by young people throughout lockdown periods could directly engage young people using methods such as photovoice or walking interviews, which can assist participants to articulate their production of public space. This study is limited through the non-participant approach to observation employed. However, this research highlights the value of studying changes to space as a means of understanding the practices occurring in the space, and the significance of the traces left behind in the landscape by young people.
